# MoleculeFormer is a GCN-transformer architecture for molecular property prediction

**DOI:** 10.1038/s42003-025-09064-x

**Published:** 2025-11-25

**Authors:** Mingyuan Qin, Ziyan Sun, Lei Feng, Chongyin Han, Jingjing Xia, Lianyi Han

**Affiliations:** 1 Department of Dermatology, Huashan Hospital, Shanghai Institute of Dermatology, Fudan University, Shanghai, China; 2 State Key Laboratory of Genetics and Development of Complex Phenotypes, School of Life Sciences, Fudan University, Shanghai, China; 3 Greater Bay Area Institute of Precision Medicine (Guangzhou), School of Life Sciences, Fudan University, Guangzhou, China

**Keywords:** Computational biology and bioinformatics, Drug discovery

## Abstract

Artificial intelligence is increasingly important in drug discovery, particularly in molecular property prediction. Graph Neural Networks can model molecular structures as graphs, using structural data to predict molecular properties and biological activities effectively. However, molecular feature optimization and model integration remain challenges. To address these challenges, we propose MoleculeFormer, a multi-scale feature integration model based on Graph Convolutional Network-Transformer architecture. It uses independent Graph Convolutional Network and Transformer modules to extract features from atom and bond graphs while incorporating rotational equivariance constraints and prior molecular fingerprints. The model captures both local and global features and introduces 3D structural information with invariance to rotation and translation. Experiments on 28 datasets show robust performance across various drug discovery tasks, including efficacy/toxicity prediction, phenotype screening, and ADME evaluation. The integration of attention mechanisms enhances interpretability, and the model demonstrates strong noise resistance, establishing MoleculeFormer as an effective, generalizable solution for molecular prediction tasks.

## Abstract (web summary)

MoleculeFormer is a GCN-Transformer architecture that integrates atomic and bond-level graphs with 3D features and molecular fingerprints, enabling comprehensive, interpretable, and accurate molecular property prediction.

## Introduction

In recent years, AI-based approaches have greatly improved the accuracy and efficiency of predicting small molecule properties. These methods utilize machine learning and deep learning techniques to dissect the relationship between molecular structures and their properties, including physicochemical properties, and other relevant characteristics of small molecules, thereby predicting biological activities.

With advancements in computational power and the widespread availability of big data, AI-based approaches can comprehensively and rapidly explore molecular information space, uncovering patterns and regularities hidden within large datasets. These methods include but are not limited to QSAR and QSPR^1,2^, which effectively handle complex relationships between molecular structures and multiple properties. Such capabilities have established AI as a transformative tool for drug discovery, material design, environmental science, and other fields.

Embedding small molecule features remains a critical step, emphasizing the importance of extracting relevant molecular characteristics as a key research direction^3^. This process includes mainly molecular representation and atomic representation. Both approaches take SMILES as input and ultimately output the feature representation of the entire molecule.

Molecular representation expresses molecular features through prior knowledge, including molecular descriptors and fingerprints. Common molecular descriptors include physicochemical descriptors, topological descriptors, and quantum chemical descriptors^4–6^. Some descriptors that have explicit relationships with specific properties may perform poorly in tasks where prior knowledge is not well-established^7^. Molecular descriptors provide detailed physicochemical information through numerical computation, while molecular fingerprints are a more structured encoding method^8^. They mainly generate a binary or hashed code by identifying structural fragments, functional groups, or substructures within molecules. The encoding logic and feature content of molecular fingerprints are different. Typically, multiple fingerprints are used to represent a molecule and thus describe its structural patterns. These fingerprint inputs are modeled using both traditional machine learning methods, such as Naive Bayes (NB)^9,10^, Support Vector Machines (SVM)^11–13^, Random Forest (RF)^14–16^, Extreme Gradient Boosting (XGBoost)^17,18^, and deep learning algorithms. Zagidullin et al.^19^ discussed 11 types of molecular fingerprints and explored how to identify the optimal molecular representation type. Molecular descriptors and fingerprints both discard some molecular structural information and heavily rely on prior knowledge.

Atomic representation operates at the atomic level, converting SMILES into graphs where atoms are nodes and bonds are edges. Atomic and bond features (such as atomic number, valence electrons, single or double bonds) are embedded. Node representations are updated by aggregating neighboring node information, capturing the structure of the graph. Graph convolutional neural networks (GCNs) aggregate features through convolution operations on graph structures, updating representations of each node^20,21^. Graph attention networks (GATs) assign different importance weights to neighbors of each node, considering the influence of different neighbors during feature aggregation^22^. Graph Transformers interpret self-attention as a soft adjacency matrix between elements of the input sequence^23^. Chemprop uses a Directed Message Passing Neural Network (D-MPNN) to extract molecular features^24^. HiGNN employs hierarchical graph convolution to capture complex structural information in molecules^25^. In the final prediction layer, to represent several atoms as the molecular feature expression while maintaining consistent dimensions, pooling is typically used. Aggregated nodes often require pooling to represent the entire graph feature. This method crudely averages or maximizes atomic features, discarding a significant amount of feature detail.

All the above models encode atomic features without considering the adjacency relationship of bonds. Bond energy not only directly affects the thermodynamic stability of molecules (such as decomposition temperature and reaction activation energy) but is also closely related to intermolecular interactions (such as hydrogen-bond strength and π-π stacking effect) and dynamic behaviors (such as vibration frequency and photochemical reaction pathways). Integrating bond energy parameters into the edge feature encoding layer, or generating bond energy-related derived descriptors through quantum chemical calculations, and incorporating such information into the scope of graph encoding can better assist the model in predicting molecular properties.

Combining graph-based representations with descriptor-based representations often leads to better model performance. Wu et al.’s HRGCN+ model presented a simple but highly efficient modeling method by combining molecular graphs and molecular descriptors as input to a modified graph neural network^26^. Cai et al. proposed the FP-GNN model^27^, which integrates three types of molecular fingerprints with graph attention networks, further enhancing the model’s performance and interpretability.

This study proposes an interpretable molecular prediction model based on multi-scale features. In terms of graph encoding selection, instead of the traditional single-molecule graph mode, we incorporate bond graph information with a feature length of 39. That is, each bond is regarded as a node, and adjacent bonds are connected. The atom graph mainly focuses on the attributes of atoms in the molecule, but its description of the relationships between atoms is relatively limited. Bond graph information can serve as an important supplement to the atom graph, accurately describing the bonding situations between atoms, including bond types (such as single bonds, double bonds, and triple bonds), bond lengths, and bond angles. These details are crucial for understanding the geometric shape and chemical properties of molecules. For example, in organic chemistry, the presence of double and triple bonds can significantly affect the reaction activity and spatial configuration of molecules. By incorporating bond graph information, the model can more comprehensively capture the structural features of molecules, thereby improving the prediction accuracy of molecular properties and behaviors.

In the graph aggregation aspect, traditional graph models such as GCN^21^ and GAT^22^ use node aggregation. They transfer information between a node and its neighboring nodes and finally use operations like max-pooling and average-pooling to compress the features of all nodes and extract graph features. This approach leads to a large amount of information loss, and it is difficult to explain from a model perspective whether the pooling operations are effective. Inspired by natural language processing (NLP), we use the GCN model to replace positional encoding and the Transformer encoder model to extract features by treating the molecular graph as a sentence. Therefore, we introduce graph-representation nodes. By analyzing the correlation between the graph-representation node and each node through the model, we achieve feature clustering of the entire graph. Moreover, this approach can be reliably explained by combining the graph representation with the attention weights of each node, allowing us to analyze which part of the molecule has a greater impact on its properties.

During the representation and aggregation stages, we introduce three-dimensional (3D) features for encoding. Meanwhile, our model integrates Equivariant Graph Neural Networks (EGNN)^28^, maintaining rotational and translational variability. Its equivariant update ability also makes the node update rule equivariant. This means that when updating node features, the influence of geometric transformations is taken into account. When updating the 3D position features of atoms, the calculation is based on the current position of the atom and the position information of its neighboring atoms, while keeping the distances between adjacent atoms unchanged.

**Fig. 1:**
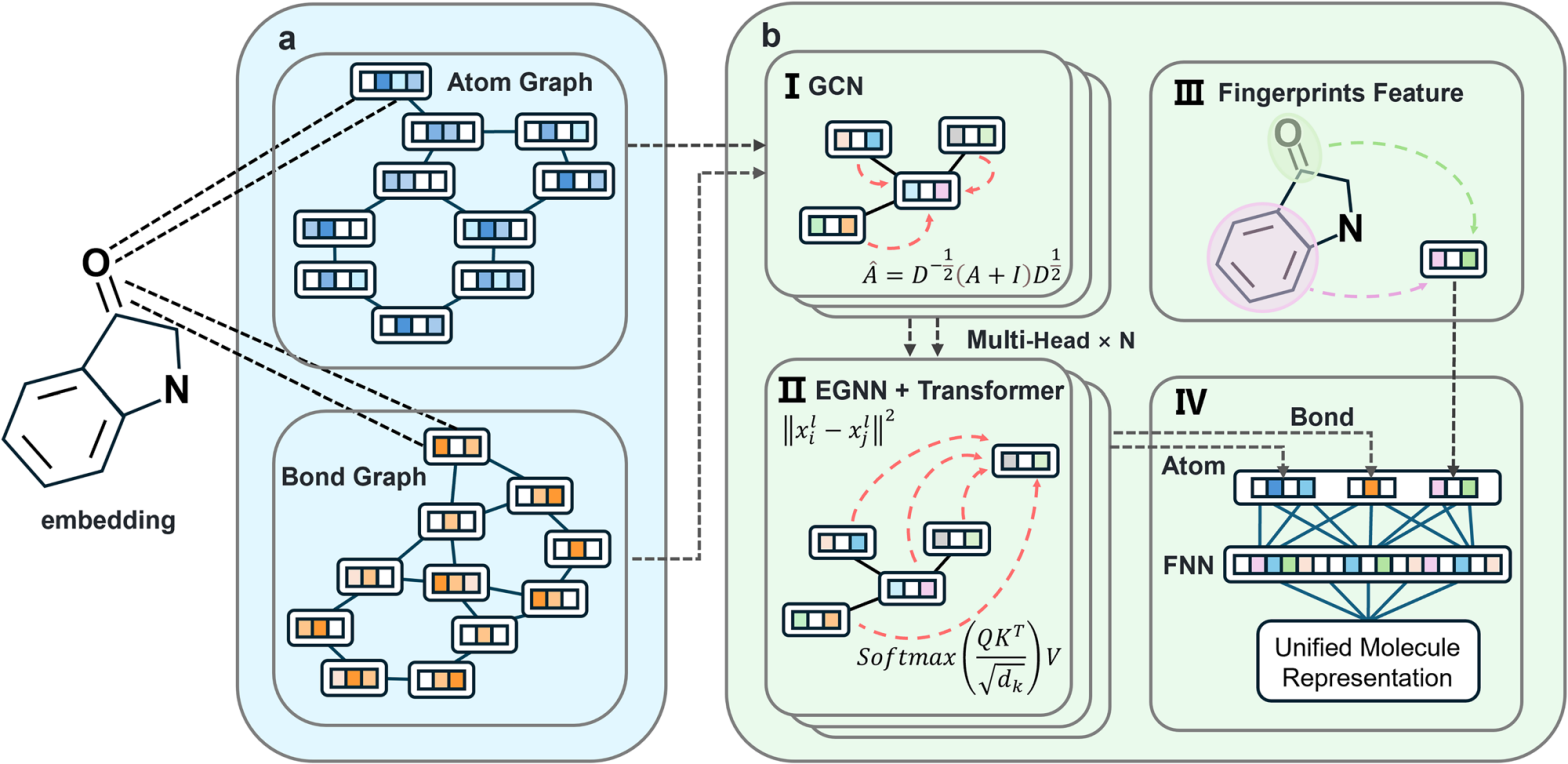
The architecture of MoleculeFormer. **a** Convert the SMILES embeddings into an atom graph and a bond graph, respectively. **b** GCN-Transformer Equivariant Encoder and output head. **I** Use GCN for aggregation to transform the graph into a graph structure with local information. **II** Perform Transformer encoding with EGNN to output molecular features. **III** Incorporate prior knowledge into the molecular fingerprint encoding. **IV** Use FNN to output the prediction results.

Through this approach, our model, while satisfying graph equivariance, employs a multi-dimensional interpretable design. By using the attention mechanism and combining the molecular feature extraction of atomic graphs and bond graphs, it realizes the visual presentation of molecular structure attention at the microscopic level. Meanwhile, it incorporates molecular fingerprint encoding with prior knowledge to ensure the accuracy and fitting speed of the model (Fig. 1). This multi-dimensional molecular property extraction model maximally extracts the molecular features to guarantee the reliability and accuracy of practical tasks.

## Results

### Selection of combinations of molecular fingerprints

To identify the best molecular fingerprints, we systematically evaluated the performance of eight commonly used molecular fingerprints^29–36^ in cheminformatics tasks based on three dimensions: authority, encoding completeness, and widespread usage. To ensure the rigor of the experimental design, we employed a controlled variable approach, conducting separate encoding selection experiments for single, double, and triple fingerprints. Specifically, for each combination, we constructed identical Feedforward Neural Network (FNN) model architectures, adjusting only the input layer dimensions to accommodate the lengths of different fingerprint combinations, thereby ensuring that performance differences stem solely from the fingerprint selection itself. We noticed a significant correlation between the selection of molecular fingerprints and the task type. To thoroughly explore this correlation, we conducted detailed statistical analyses on regression tasks and classification tasks separately. Among them, the classification tasks included 7 classification tasks from the MoleculeNet dataset^37^ and 14 classification tasks from the breast cancer dataset^38^. The regression tasks consisted of 3 datasets from the MoleculeNet and 4 datasets from the ADME dataset^39^.

**Fig. 2:**
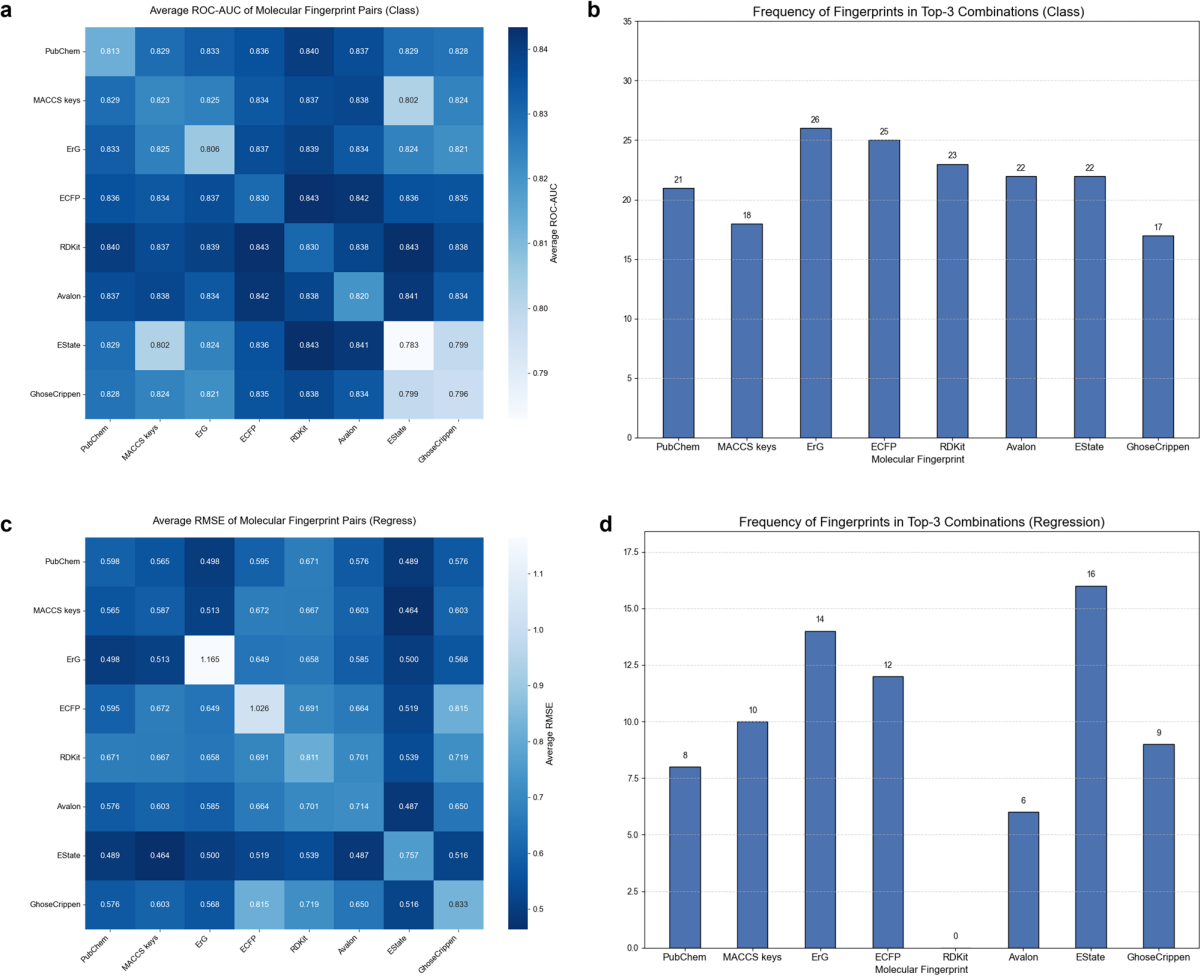
The results of the molecular fingerprint selection experiment. Experiments were carried out using combinations of 1, 2, and 3 molecular fingerprints, respectively. **a** Heatmap of two-molecule fingerprint combinations in the classification task. **b** Statistics on the counts of molecular fingerprints in the top 3 combinations of each dataset in the classification task. **c**, **d** Results for the regression task (heatmap and statistical counts, respectively; corresponding to **a** and **b**). In heatmaps, darker colors indicate better performance metrics. Compared with the classification task, molecular fingerprints showed greater differences in the regression task. The EState fingerprint ranked fourth and last among single-molecule fingerprints in both tasks, respectively, performing worse than other fingerprints. However, it demonstrated excellent performance when used in combinations, which reflects the importance of the complementarity of fingerprint features. The RDKit fingerprint had a worse interfering effect in the regression task.

The experimental results (Fig. 2) showed that there were obvious differences in the performance of single-molecule fingerprints for different tasks. In the classification tasks, ECFP fingerprint and RDKit fingerprint stood out, achieving an excellent average AUC of 0.830. This is because the core of classification tasks is to accurately distinguish different classes of molecules. ECFP fingerprint can meticulously describe the local structure and atomic environment of molecules, which is crucial for identifying the characteristic differences of molecules in different classes. In contrast, in the regression tasks, the MACCS keys performed the best, with an average RMSE of 0.587. The goal of regression tasks is to predict continuous values, such as certain physical or chemical properties of molecules. The MACCS keys fingerprint may contain information closely related to these continuous properties, and its encoding rules can effectively reflect the linear or non-linear relationships between some key features of molecules and the target values.

In the experiments of combining two molecular fingerprints, the optimal combinations also varied for different tasks. In the classification tasks, the optimal combination was still ECFP fingerprint and RDKit fingerprint, with an average AUC remaining at 0.843. This indicates that these two fingerprints are highly complementary in providing features required for classification. The feature information they contain can complement each other, enabling the model to distinguish different molecular classes more accurately. Combinations with the same prediction efficacy also include the EState fingerprint and the RDKit fingerprint. When the EState fingerprint is used alone, its average AUC (0.783) is significantly lower than that of the optimal combination, indicating that the ECFP fingerprint has more effective features than the EState fingerprint in classification tasks. In the regression tasks, the performance of the combined molecular fingerprints was significantly improved. The MACCS keys + EState fingerprint combination performed the best, with the average RMSE dropping to 0.464. The EState fingerprint emphasizes the electronic state and atomic environment of molecules. Regression tasks require a more comprehensive description of various properties of molecules to accurately predict continuous values. The information contained in these two fingerprints complements each other precisely. One focuses on the relationship between some key features and the target values, and the other focuses on aspects such as the electronic state and atomic environment, jointly providing richer and more comprehensive feature information for the regression model and thus significantly improving the prediction performance.

To select the three molecular fingerprints with the best average performance in different datasets, we counted the molecular fingerprints used in the top-three combinations in each dataset and obtained the average performance rankings of molecular fingerprints in classification tasks and regression tasks. In the classification tasks, the performance of each model was relatively balanced, and the optimal combination was ErG, ECFP, and RDKit. ErG fingerprint focuses on pharmacophore-related information and can reflect the characteristics of molecules related to biological activity. ECFP fingerprint is based on the substructures around atoms and can reflect the overall atomic environment characteristics of molecules. RDKit fingerprint extracts information from the perspective of molecular graph paths and can capture the local bond characteristics of molecules. Using them in combination can more comprehensively describe the molecular structure and properties. Classification models require multi-dimensional features to accurately distinguish different types of molecules. Comprehensive utilization of these three types of fingerprints can provide rich and comprehensive features for classification models, improving the accuracy and reliability of classification.

In the regression tasks, the EState fingerprint performed the most prominently, while the RDKit fingerprint performed poorly and did not make it into the rankings. EState fingerprint starts from the electronic and topological properties of atoms and reflects the intrinsic state of atoms in molecules. Many continuous physical and chemical properties, such as the polarity and reactivity of molecules, are closely related to the electronic and topological environment of atoms. For example, the solubility of a molecule is related to the charge distribution and spatial arrangement of atoms within the molecule, and the EState fingerprint can capture such atomic-level information, thus better establishing a quantitative relationship between the molecular structure and continuous properties such as solubility.

It is worth mentioning that in the single-molecule fingerprint prediction tasks, ErG and ECFP were the two worst-performing fingerprints, but they ranked among the top three in the molecular rankings of the regression model. This phenomenon indicates that ErG and ECFP can serve as excellent supplementary information for other molecular fingerprints, providing additional feature dimensions for the model. Therefore, EState fingerprint, ERG fingerprint, and ECFP fingerprint are used as the fingerprints for regression models.

### Performance of the MoleculeFormer architecture on the MoleculeNet datasets

Public benchmark datasets from Wu et al.^37^ were used to evaluate the performance of the MoleculeFormer across 10 widely recognized benchmark datasets for drug property prediction. The tasks include both classification and regression, with datasets split either randomly or by scaffold. Encompassing both single-task and multi-task datasets, these benchmarks are highly representative and extensively studied in drug discovery, offering significant evaluation value.

We used two methods, random splitting and scaffold-based splitting, to divide the datasets. Each dataset was randomly partitioned into a training set, a validation set, and a test set at an 8:1:1 ratio. Each experiment used 10 different random number seeds to ensure the robustness and generalizability of the results. ROC-AUC and PRC-AUC were used as the evaluation metrics for all datasets, where PRC-AUC is particularly suitable for imbalanced datasets or tasks focusing on minority classes. In regression tasks, RMSE (Root Mean Square Error) is a metric for measuring the accuracy of a regression model, representing the square root of the average of the squared differences between predicted values and actual values.

As shown in Table 1, the MoleculeFormer model achieved the best average performance across 13 tasks, demonstrating its effectiveness. Specifically, the BACE dataset^40^ is used for drug design and molecular docking studies, predicting the binding affinity between molecules and the β-secretase enzyme (BACE); the HIV dataset^41^ is typically used to predict the inhibitory activity of compounds against Human Immunodeficiency Virus (HIV); the BBBP dataset^42^ predicts compound permeability across the Blood-Brain Barrier (BBB), which is crucial for drug screening for central nervous system; the Tox21^43^ and the ClinTox^44^ datasets predict drug toxicity status; the SIDER dataset^45^ predicts relationships between drugs and side effects, covering various adverse reactions caused by medications; the MUV dataset^46^ (Most Useful Variants) evaluates the predictive performance of compounds interacting with target proteins; the FreeSolv dataset is used to predict compound solubility^47^; the ESOL dataset assesses the accuracy of predictions for the water solubility of compounds (logP)^48^ and the Lipophilicity dataset predicts the lip-water partition coefficient (log D) of compounds^49^.

**Table 1:** Predictive performance results of MoleculeFormer on 10 commonly used public datasets

Dataset	Split type	Metric	MoleculeNet (Graph)	Chemprop (optimized)	Attentive FP	HRGCN+	XGBoost	FP-GNN	MoleculeFormer
BACE	Random	ROC-AUC		0.898	0.876	0.891	0.889	0.881	**0.913**
	Scaffold	ROC-AUC	0.806	0.857				0.860	**0.874**
HIV	Random	ROC-AUC		0.827	0.822	0.824	0.816	0.825	**0.837**
	Scaffold	ROC-AUC	0.763	0.794				0.824	**0.830**
BBBP	Random	ROC-AUC			0.887	0.926	0.926	**0.935**	0.932
	Scaffold	ROC-AUC	0.690	0.886				0.916	**0.924**
Tox21	Random	ROC-AUC	0.832	**0.897**	0.852	0.848	0.836	0.815	0.839
ClinTox	Random	ROC-AUC	0.832	0.897	0.904	0.899	**0.911**	0.840	0.883
SIDER	Random	ROC-AUC	0.638	0.658	0.623	0.641	0.642	0.661	**0.707**
MUV	Random	PRC-AUC	0.109	0.053	0.038	0.082	0.068	0.09	**0.144**
FreeSolv	Random	RMSE	1.150	1.009	1.091	0.926	1.025	**0.905**	1.022
ESOL	Random	RMSE	0.580	0.587	0.587	**0.563**	0.582	0.675	0.645
Lipophileicity	Random	RMSE	0.655	0.563	**0.553**	0.603	0.574	0.625	0.649

Each dataset is split into training, validation, and test sets with a ratio of 8:1:1. The bold font indicates the top model with the highest scores. Attentive FP and HRGCN+ results are from Wu et al.^26^. FP-GNN results are from Cai et al.^27^.

MoleculeFormer demonstrated excellent performance in the binary classification tasks of the BACE and BBBP datasets. Specifically, on the BACE dataset, it achieved a PR-AUC of 0.87 ± 0.04. On the BBBP dataset, the PR-AUC reached 0.97 ± 0.01. The average PR-AUC on the ClinTox dataset was 0.98 ± 0.01. For the SIDER dataset (with a PR-AUC of 0.69 ± 0.02), the HIV dataset (with a PR-AUC of 0.41 ± 0.15), the Tox21 dataset, and the MUV dataset, the PR-AUC values were relatively low. This indicates that the overall classification difficulty is high on these three datasets, and there may be issues such as imbalanced sample classes and complex and diverse features. However, MoleculeFormer has more advantages compared to other models.

Notably, even though the relatively small number of training molecules in the three regression tasks limited the full functionality of MoleculeFormer’s attention mechanism, the model still maintained strong competitiveness-particularly in its performance on the datasets.

**Fig. 3:**
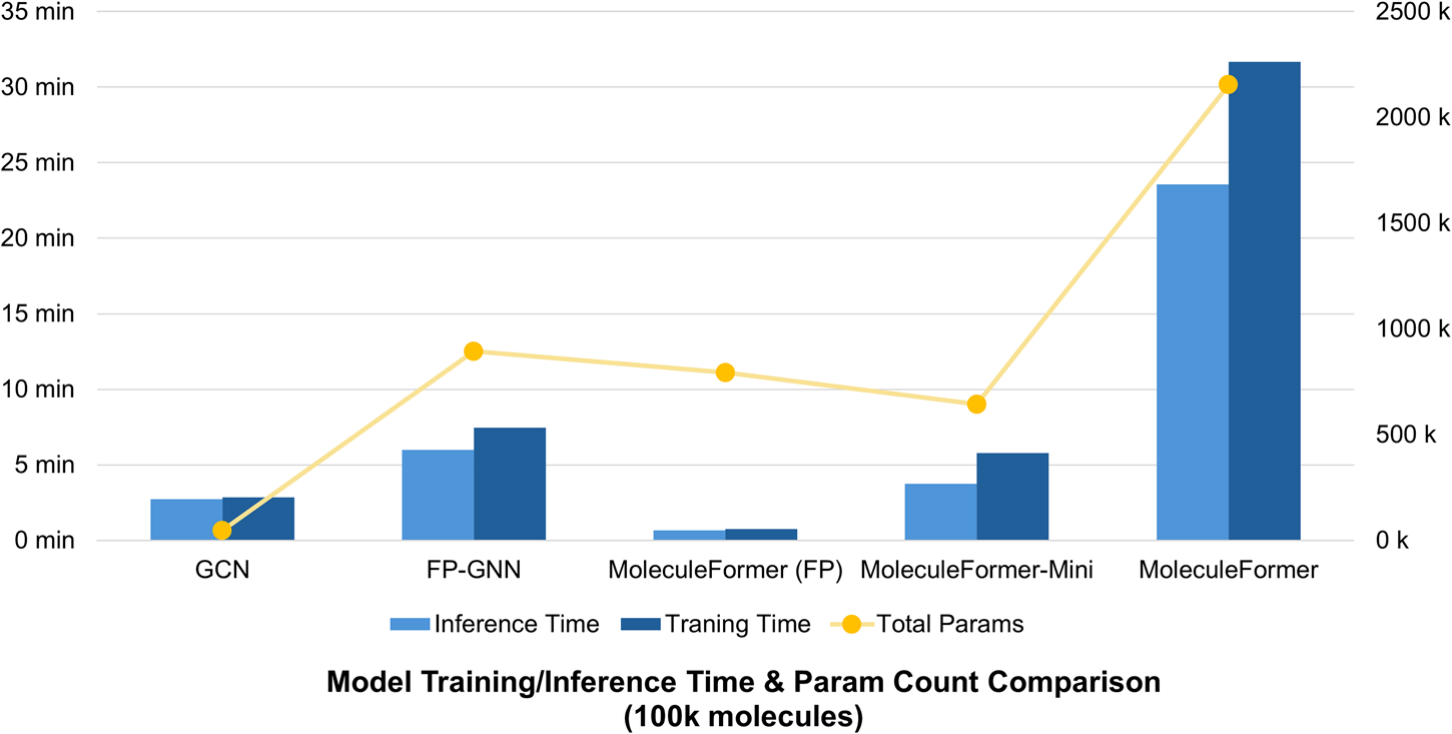
Comparison of training/inference time and parameters for models predicting 100k small molecules. A comparison chart of the training and inference time consumption and the number of parameters of different models under the training of 100k small molecules, excluding the time consumption for generating 3D features of small molecules and force field optimization. To address the issue of excessively long training time of the model with large-scale data, we also developed the MoleculeFormer-Mini version. Compared with MoleculeFormer, this version sacrifices a small amount of accuracy, and it takes less time than FP-GNN.

Figure 3 shows the time required for training and inference of different models after inputting information of 100k molecules (including 3D information) and their number of parameters. MoleculeFormer (FP) directly uses molecular fingerprints for fully connected encoding, so its training and inference are relatively fast. Compared with MoleculeFormer, MoleculeFormer-Mini does not have EGNN, which saves a significant amount of training time while still retaining the GCN and Transformer modules.

### Performance of the MoleculeFormer architecture on the cell-based phenotypic screening datasets

To evaluate whether MoleculeFormer can capture the holistic effects of drugs and assess their comprehensive impact on real cells, we conducted experiments using a compound-breast cancer cell phenotypic screening dataset. This dataset, curated by He et al.^38^, aggregates all available quantitative compound-cell interaction data for 13 breast cancer (BC) cell lines and 1 normal breast cell line. Compounds were retained only if their activity was quantified via standard metrics (IC50, EC50, or GI50); molecules without validated activity records were excluded. Applying established thresholds from Fields et al.^50^ and Ye et al.^51^, compounds with activity values ≤ 10 μM were classified as active, while those >10 μM were labeled inactive; unclassifiable cases were discarded. The final dataset consisted of 33,757 active and 21,152 inactive compounds, all commonly used in in vitro anti-proliferation assays.

We evaluated MoleculeFormer on the aforementioned 14-cell-line dataset (Table 2). For comparison, we included published results from He et al.^38^, which encompassed five graph-based deep learning models: the Attentive FP^52^ based on the graph attention mechanism, a model based on the message passing neural network (MPNN)^53^, an advanced fingerprint-based XGBoost^54^ model, the FP-GNN^27^ model, and a fingerprint module from the MoleculeFormer. MoleculeFormer was trained and tested on the same phenotypic screening data, with performance metrics directly compared to these reference models. As summarized in Table 2, top-performing models for each cell line are highlighted in bold. Notably, MoleculeFormer achieved the highest average ROC-AUC score across all cell lines, demonstrating its competitive predictive accuracy in this task.

**Table 2:** Predictive ROC-AUC performance results of MoleculeFormer on 14 breast cell line datasets compared to the graph-based DL models

Cell lines	Classification	Compounds	Attentive FP	MPNN	XGBoost	FP-GNN	MoleculeFormer (FP)	MoleculeFormer
MDA-MB-453	HER2+^a^	440	0.872	0.715	0.810	**0.886**	0.838	0.866
SK-BR-3	HER2+	2026	0.805	0.760	0.848	0.852	**0.874**	0.858
MDA-MB-435	HER2+	3030	0.824	0.749	**0.853**	0.820	0.842	0.847
T-47D	Luminal A^b^	3135	0.812	0.751	0.821	0.846	0.844	**0.858**
MCF-7	Luminal A	29378	0.845	0.843	0.826	0.866	0.837	**0.875**
MDA-MB-361	Luminal B^c^	367	0.938	0.972	**0.976**	0.905	0.91	0.922
BT-474	Luminal B	811	0.787	0.847	0.827	0.868	0.896	**0.906**
BT-20	TNBC^d^	292	0.735	0.784	0.740	**0.887**	0.805	0.833
BT-549	TNBC	1182	0.630	0.634	0.651	0.807	0.811	**0.825**
HS-578 T	TNBC	469	**0.830**	0.665	0.753	0.770	0.804	0.813
MDA-MB-231	TNBC	11202	0.870	0.850	0.865	0.866	0.852	**0.871**
MDA-MB-468	TNBC	1986	0.875	0.858	**0.896**	0.888	0.868	0.886
Bcap37	TNBC	275	**0.858**	0.807	0.744	0.779	0.748	0.796
HBL-100	Normal cell line	316	0.645	0.701	0.776	0.850	0.820	**0.914**
Average			0.809	0.781	0.813	0.849	0.839	**0.862**

^a^HER2 + : HER2-positive breast cancers. ^b^Luminal A: Luminal A breast cancer is hormone-receptor positive (estrogen-receptor and/or progesterone-receptor positive), HER2 negative, with low levels of the protein Ki-67. ^c^Luminal B: Luminal B breast cancer is hormone-receptor positive (estrogen-receptor and/or progesterone-receptor positive), HER2 positive or HER2 negative, with high levels of Ki-67. ^d^TNBC: triple-negative breast cancer. Each dataset was split into training, validation, and test sets using the corresponding data-split codes from He et al.^38^ and Cai et al.^27^. The MoleculeFormer model used the same datasets and data-split method to fairly compare the Attentive FP^55^, MPNN^24^, XGBoost^54^, and FP-GNN^27^ models. Meanwhile, to verify the advantages of information fusion between GNN and Fingerprint, we separately extracted the molecular fingerprint module from the MoleculeFormer model for prediction. The bold font indicates the top model with the highest scores. *MPNN* message passing neural networks.

### Ablation study of MoleculeFormer

In drug development, the absorption, distribution, metabolism, and excretion (ADME) properties of drug molecules directly determine efficacy and safety. The Biogen dataset^39^ used in this study focuses on ADME characteristics of commercially available compounds, encompassing structurally diverse molecules that simulate the complexity of real drug candidates. This dataset enables the evaluation of the ability of MoleculeFormer to learn broad molecular features while quantifying the contribution of each module to predictive performance.

In the ablation study, we compared six model architectures: a baseline Graph Convolutional Network (GCN), an atom graph-based Transformer model (Atom), an atom graph + bond graph joint Transformer model (Atom + Bond), a molecular fingerprint (FP) encoding model, an atom graph + bond graph joint Transformer model with fingerprint (Atom + Bond + FP), and a composite model integrating atom graph, bond graph, FP, and EGNN modules, namely MoleculeFormer. The experiments employed RMSE as the evaluation metric, with fairness ensured by fixing 10 random split seeds across all datasets (Table 3). Key findings include:

1. Synergy of Atom and Bond Graphs: The fusion of atom and bond graphs enhanced the extraction of implicit molecular features while improving prediction stability.
2. Complementarity of Multimodal Features: Incorporating molecular fingerprints strengthened the model’s representational capability, demonstrating complementary effects with graph-based architectures to construct a more comprehensive molecular representation.
3. 3D Feature Sensitivity: In datasets involving molecular conformations or spatial dependencies, the integration of the EGNN module significantly improved predictive performance, highlighting its advantage in capturing 3D structural features.

**Table 3:** The ablation study of the MoleculeFormer model for 4 different datasets using RMSE as the evaluation metric

RMSE	GCN	Atom	Atom + Bond	FP	Atom + Bond + FP	MoleculeFormer
HLM	0.571+/− 0.020	0.466+/− 0.017	0.468+/− 0.012	0.472+/−0.018	0.464+/− 0.021	**0.462+/−0.021**
MDR1-MDCK ER	0.640+/− 0.027	0.465+/− 0.028	0.466+/−0.018	0.463+/−0.031	0.465+/−0.018	**0.453+−0.013**
RLM	0.661+/− 0.023	0.553+/− 0.021	0.546+/− 0.013	0.545+/−0.025	0.562+/−0.018	**0.510+−0.042**
SOLUBILITY	0.636+/−0.028	0.563+/− 0.047	0.561+/− 0.038	0.562+/−0.043	**0.555+/−0.015**	0.556+/−0.045
Avgrage	0.627+/− 0.025	0.512+/− 0.028	0.510+/− 0.020	0.511+/− 0.029	0.511+/− 0.018	**0.495+/−0.030**

HLM: Logarithmic data of the intrinsic clearance (CLint) of human liver microsomes (HLM). MDR1-MDCK ER: Logarithmic data of the efflux ratio (ER) in the MDR1-MDCK cell model. RLM: Logarithmic data of the intrinsic clearance (CLint) of rat liver microsomes (RLM). SOLUBILITY: Logarithmic data of drug solubility at pH 6.8. In each ablation experiment, 10 identical random split seeds were selected from the dataset. This ensured that each model was provided with the same 10 sets of training, validation, and test datasets for experimentation. The ratio of the training, validation, and test datasets was set at 8:1:1.

### The anti-noise ability of MoleculeFormer

Deep learning models rely on the accuracy of dataset labels, as incorrect labels can cause the model to learn erroneous feature weights. However, experimental errors are unavoidable during dataset collection, making noise robustness a crucial measure of model quality.

**Fig. 4:**
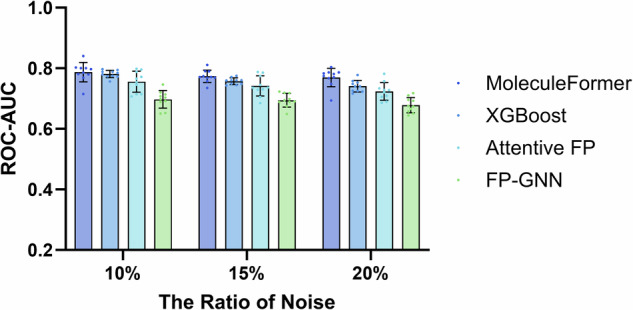
Anti-noise performance comparison across different noise rates. The anti-noise performances of Attentive FP, XGBoost, FP-GNN, and MoleculeFormer with different noise rates on the HIV dataset were evaluated. The FP-GNN model was sourced from Cai et al.^27^. The Attentive FP model was sourced from Xiong et al.^52^. XGBoost employed the same molecular fingerprint selection as MoleculeFormer. Each experiment included 10 independent samples, and all experiments were conducted without interference between them. Error bars indicate mean ± standard error.

We conducted experiments using the MoleculeFormer model on the HIV dataset, where a portion of the labels in the training and validation sets were reversed, and experiments were performed using 10 random number seeds. We compared the results with noise experiments conducted under the same conditions using Wu et al.’s Attentive FP^55^, Cai et al.’s FP-GNN model^27^, and 10 independent experiments with the eXtreme Gradient Boosting algorithm using ErG, ECFP, and RDKit molecular fingerprints. Figure 4 shows that our MoleculeFormer model exhibits outstanding noise robustness, indicating that it performs well even with lower-quality datasets (Supplementary Table 3).

### The interpretation of MoleculeFormer

To assess the interpretability of MoleculeFormer, we employed the BBBP dataset. The dataset underwent two-stage cleaning: (1) removal of 74 duplicate small molecules to ensure data uniqueness, and (2) exclusion of 4 invalid SMILES structures to maintain data integrity. We then retrained the model on this refined dataset and conducted interpretability experiments. Xiong et al.^56^ reviewed recent studies aimed at modifying small molecules to increase brain exposure and summarized strategies used by medicinal chemists to enhance the BBB permeability through structural modifications of small molecules, including improving lipophilicity and reducing hydrogen-bond donor capacity, to facilitate the passage of small molecules through the BBB. The prediction results of the MoleculeFormer are consistent with the relevant feature results from laboratory experiments, demonstrating that our model can effectively extract relevant features and use them as the basis for judgments.

### Lipophilicity

Lipophilicity refers to the affinity of a compound for lipids or nonpolar solvents, while calculated LogP (cLogP) is an indicator used to estimate the distribution ratio of a compound between oil and water, reflecting its lipophilicity. Enhancing the lipophilicity of a molecule can improve its permeability across the BBB.

**Fig. 5:**
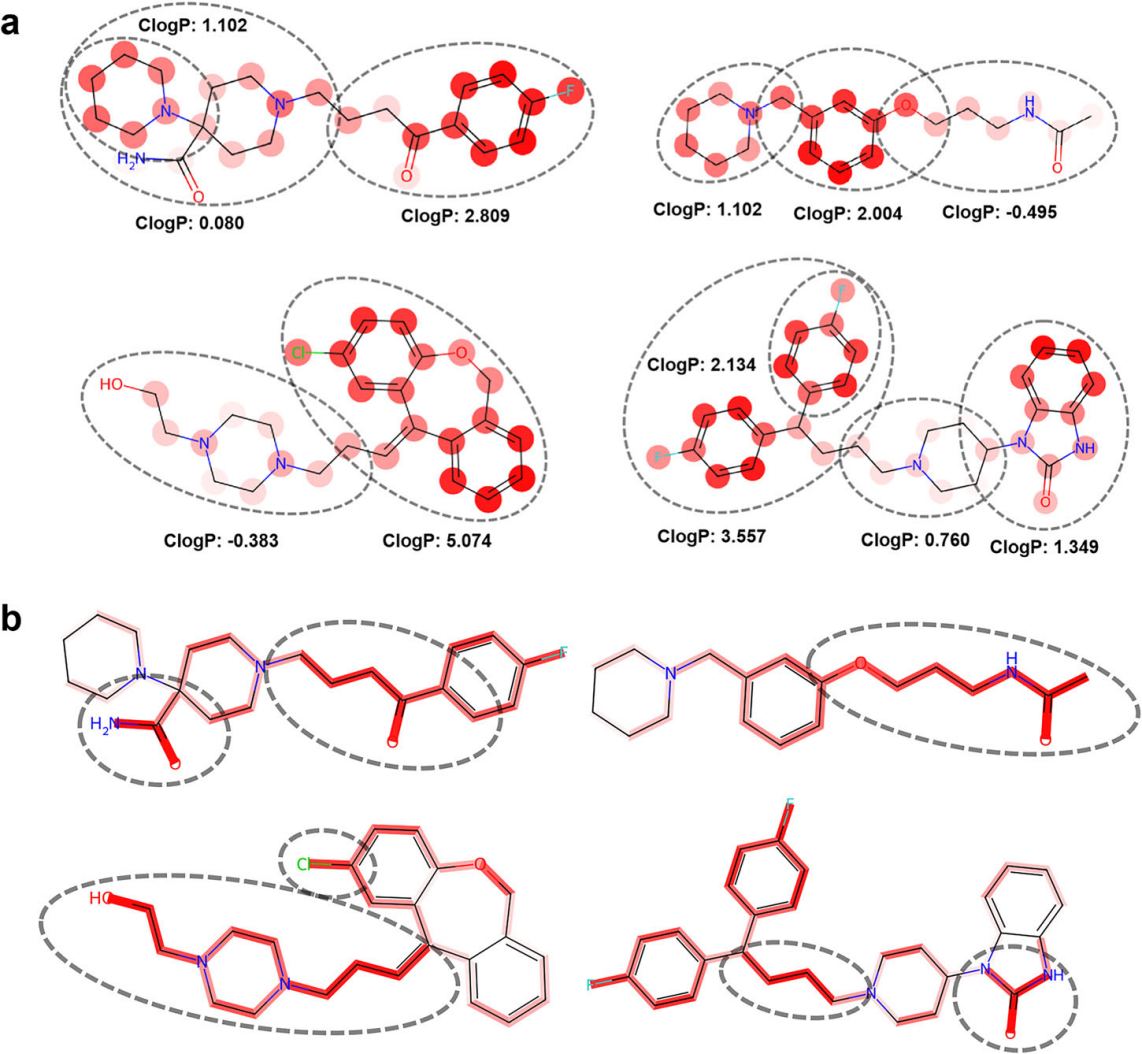
Visualization of attention weights in the transformer layer. Each molecule is divided into two parts, with cLogP values calculated separately for each. Additionally, we visualized the attention weights in the Transformer layer, where the shade of color represents the attention weight of each atom, with darker colors indicating higher weights. Our model pays more attention to regions with higher cLogP values (**a**). The bond graph pays more attention to polar groups, thus affecting the HBD of the molecule. The carbon chain structure offers lower 3D rigidity, which is beneficial for passing through the pore size limit of the BBB (**b**).

In the study of the atomic attention mechanism of MoleculeFormer, the model quantifies the relative importance of each atom within the molecule through the self-attention layer and reveals its decision-making basis with the help of visualization technology. As shown in Fig. 5a, the attention weights at the atomic level are presented through color gradients. The dark-colored regions correspond to features with high cLogP values (such as hydrophobic groups, aromatic ring substituents, etc.), while the light-colored regions represent structures with low cLogP values. Notably, the model shows systematic attention to high cLogP value regions, which is mechanistically consistent with the theory proposed by Xiong et al. that “enhancing lipophilicity can improve the BBB permeability”.

Specifically, in molecules containing long alkyl chains or halogen substituents, the attention weights are significantly concentrated on these highly lipophilic structural domains. Experimental evidence shows that such groups can effectively promote the transmembrane diffusion process by increasing the molecular lipid partition coefficient. This attention distribution pattern not only verifies that the model can accurately capture the chemical features closely related to the BBB permeability but also explains the mechanism by which lipophilic groups improve drug delivery efficiency from a computational perspective. By transforming the abstract feature extraction process into an interpretable visual representation, this study confirms that the decision-making logic of MoleculeFormer is highly consistent with experimental chemical intuition, thus providing reliable theoretical support for its prediction results.

**Fig. 6:**
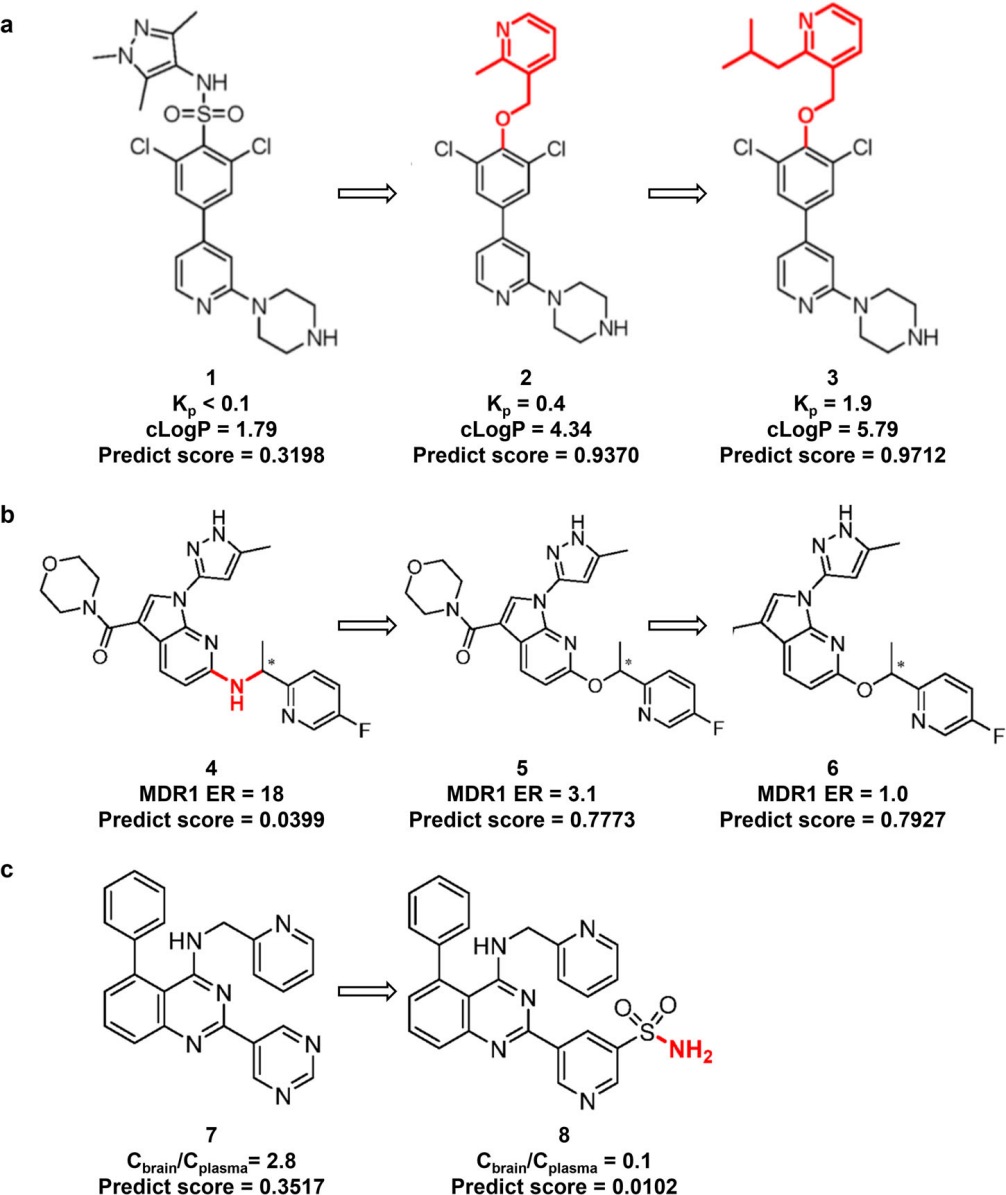
Optimization of BBB penetration of compounds and validation of model prediction consistency. Brand et al. performed two rounds of optimization on the poorly BBB-permeable compound 1. Higher Kp and cLogP values indicate better BBB penetration^53^ (**a**). Fushimi et al. performed two rounds of optimization on the poorly BBB-permeable compound 4. MDR1 (P-glycoprotein) is a transport protein in the BBB that actively pumps out drugs, thereby reducing their ability to penetrate the brain. Lower MDR1 levels correspond to stronger BBB penetration^58^ (**b**). Gunaga et al. reduced the risk of BBB penetration of compound 7, which had the potential to cross the barrier. A lower Cbrain/Cplasma ratio indicates weaker BBB penetration^59^ (**c**). The predictions from our MoleculeFormer were consistent with the actual results, with prediction scores ranging from 0 to 1.

Brand et al.^57^ developed the lead compound 1 (DDD85646) through optimization of a high-throughput screening hit. Compound 1 exhibited potent activity against N-myristoyltransferase (NMT), a promising drug target for human African trypanosomiasis (HAT) caused by the parasitic protozoa Trypanosoma brucei. However, compound 3 had poor BBB penetration (Kp < 0.1). To improve its BBB penetration, Wyatt and colleagues conducted two rounds of optimization: they first designed and synthesized compound 2, which significantly enhanced penetration; then they further improved the lipophilicity of the compound and synthesized compound 3, which showed even better brain penetration levels (Fig. 6a). We subjected these three compounds to a predictive model. The results indicated that compound 1 had low predicted penetration capability, while compounds 2 and 3 demonstrated progressively enhanced penetration capabilities. These predictions were consistent with the actual experimental results, showing agreement between the model and empirical findings.

### Hydrogen-bond donor capacity

Research has confirmed that hydrogen-bond donor capacity (HBD) is one of the most critical factors affecting the BBB permeability, with reduced HBD generally leading to improved BBB penetration. Modifying HBD is also a common strategy in drug design.

Through the bond attention weight experiment (Fig. 5b), it was found that bond attention focuses more on the polar bonds in the molecule, and polar bonds can affect the HBD of the molecule. The carbon chain structure has lower 3D rigidity, which is beneficial for molecules to pass through the pore size limit of the BBB. The functional group structure may reflect its sensitivity to the HBD ability. HBD can significantly enhance the interaction between the molecule and the polar groups in the BBB membrane, thus hindering the passive diffusion of the molecule. Carbon chain and non-cyclic structures usually have a smaller molecular weight (MW) and lower 3D rigidity, which are beneficial for passing through the pore size limit of the BBB (generally requiring MW < 500 Da). Macrocyclic or fused-ring structures may be regarded as “obstructive features” by the model due to their large volume or high rigidity. The focus of bond attention on the molecular skeleton indicates that the model not only relies on local lipophilic features but also can learn complex structure-activity relationship rules through global structural information, further confirming the rationality of its multi-scale feature extraction ability.

Fushimi et al.^58^ explored compound 4 and enhanced its BBB penetration by removing unnecessary HBD to create compound 5. Subsequent modifications, including shielding the oxygen atom on the morpholine ring, led to compound 6, which showed further improved BBB permeability. We predicted the BBB penetration of these three compounds using a model. The results indicated that compound 4 was predicted to have no penetration capability, compound 5 showed a significant increase in predicted score, and compound 6 had an even higher predicted score, consistent with experimental improvements. The model predictions align well with the experimental results (Fig. 6b).

Gunaga et al.^59^ studied 5-phenyl-N-(pyridin-2-ylmethyl)-2-(pyrimidin-5-yl)quinazolin-4-amine (compound 7), a potential safe drug for maintaining normal sinus rhythm. However, it poses a risk of penetrating the BBB. After introducing HBD to create variant compound 8, the BBB penetration was reduced while preserving the drug’s efficacy. Our model predictions showed a significant decrease in the predicted score for the improved compound 8. The model predictions align well with the experimental results (Fig. 6c).

These findings demonstrate that MoleculeFormer exhibits strong interpretability by successfully identifying and quantifying the critical molecular features (lipophilicity and hydrogen-bond donor capacity) that govern BBB permeability-features that align with established medicinal chemistry principles. This interpretability makes the model valuable for practical applications, as it not only predicts BBB penetration but also provides chemically intuitive explanations that can directly guide molecular design in central nervous system (CNS) drug development.

## Discussion

In this study, we propose an interpretable molecular prediction model based on multi-scale features. The model employs a GCN-Transformer as the basic architecture, extracting features from atomic graphs and bond-level graphs, respectively. It dynamically captures the long-range interactions between atoms within a molecule through global attention weights in the atomic graph channel, while the bond graph channel focuses on the topological features and electronic effects of local chemical bonds. This collaborative mechanism not only enhances the model’s ability to analyze multi-level features of molecules but also reveals action mechanisms, such as key groups and implicit features in molecular prediction tasks, through the visualization of attention distributions. Furthermore, the model takes into account both the rotational equivariance of molecular graphs and the prior knowledge embedding of molecular fingerprints, and performs excellently in the collaborative extraction of local and global features of molecules.

We systematically selected multi-dimensional datasets covering molecular properties in key stages of drug development to verify the universality of the model in molecular prediction tasks. The experimental datasets include:

1. Ten core subsets in the MoleculeNet benchmark library, such as HIV (antiviral activity), BBBP (blood-brain barrier penetration), Tox21 (compound toxicity), and SIDER (drug side effects), which cover multi-scale evaluations of molecular physicochemical properties, biological activities, and safety.
2. Fourteen phenotypic screening datasets, which reflect the activity responses of molecules at the biological system level.
3. ADME pharmacokinetic datasets, which simulate the entire process of drug absorption, distribution, metabolism, and excretion in the body.

This hierarchical design not only includes classification and regression tasks but also spans the entire drug development chain from early molecular screening to late-stage efficacy evaluation. Through comparative and ablation experiments, it is shown that the model integrates different encoding models in a complementary way, demonstrating stable and excellent performance in cross-domain and multi-task molecular prediction. This confirms its generalization ability to handle complex molecular representations and multi-objective predictions.

We observed a significant correlation between the selection of molecular fingerprints and the task type, with optimal fingerprint selection being highly task-dependent. In classification tasks, ECFP and RDKit fingerprints delivered superior performance owing to their ability to capture discriminative structural differences; their combined use or integration with ErG fingerprints provided complementary structural information for optimal classification. Conversely, MACCS keys demonstrated the best single performance in regression tasks, while the combination of MACCS keys and EState fingerprints significantly enhanced continuous value prediction by integrating key feature-target relationships with electronic/topological environment descriptors. Task characteristics dictate effective fingerprints: classification relies on discriminative structural features, whereas regression necessitates descriptors correlating with continuous properties. Notably, while ErG and ECFP performed poorly as single fingerprints in regression, they substantially improved predictive capability when supplementing EState in combinations (recommended regression fingerprint set: EState + ErG + ECFP), underscoring the importance of combinatorial strategies in leveraging fingerprint complementarity.

## Methods

### Graph feature encoding

The Graph Feature Encoding module takes SMILES as input. After optimization using the general force field, it is converted into an atom graph ${G}_{A}$ and a bond graph ${G}_{B}$. The adjacency matrix of ${G}_{A}$ has a size of $N\times N$, and that of ${G}_{B}$ has a size of *M×M*, which record the information of adjacent atoms and bonds, respectively. The sizes of the atom feature matrix and the bond feature matrix are *N×F*_*1*_ and *M×F*_2_, respectively.

Here, *N* is the number of atoms in the molecule, and *M* is the number of bonds in the molecule. The atom feature *F*_1_ includes information such as atomic number, chirality, and aromaticity, with a length of 136 (Supplementary Table 1). The bond feature ${F}_{2}$ includes information such as bond type, types of the two end-atoms, and bond length, with a length of 39 (Supplementary Table 2).

### GCN-transformer equivariant encoder

This module consists of two parts: the graph convolutional local feature extraction part (Fig. 1bⅡ) and the equivariant Transformer encoder (Fig. 1b Ⅲ). The atom graph and the bond graph are trained independently in the model.

GCN represents nodes in a graph as vectors and utilizes the connectivity between nodes for information propagation and feature learning^21^. It updates node representations through a series of graph convolution operations, similar to convolution operations in computer vision, to extract and learn features from the entire graph.

As an input to the neural network, graph convolution operations can be formulated as follows:

$${H}^{(l+1)}=\sigma (\hat{A}{H}^{(l)}{W}^{(l)})$$

Where:

- $l$ is the node feature matrix at layer $l$.
- ${H}^{(l)}$ is the feature matrix of the nodes at layer $l$, with dimensions *D×F*, where *D* is the number of nodes and *F* is the number of features per node.
- ${H}^{(l+1)}$ is the feature matrix for the nodes at layer *l+*1.
- ${W}^{(l)}$ is the weight matrix for layer *l*, with dimensions F×F, where *F* is the number of features for the next layer *l+*1.
- $\sigma$ is the activation function (e.g., ReLU).

The adjacency matrix $A$ represents the connections between nodes in the graph. To normalize this matrix, we use:

$$\hat{A}={D}^{-\frac{1}{2}}\left(A+I\right){D}^{\frac{1}{2}}$$

Where:

- $\hat{A}=A+I$ is the adjacency matrix with added self-loops, where *A* is the original adjacency matrix and *I* is the identity matrix.
- *D* is the degree matrix, a diagonal matrix where each diagonal entry *D*_ii_ is the degree of node *i* (i.e., the sum of the *i*th row in *A*).
- ${D}^{-\frac{1}{2}}$ is the inverse square root of the degree matrix.

The EGNN is a deep learning architecture designed for processing graph-structured data, particularly molecular graphs.

The feature update is carried out alternately, including equivariant graph operations and global attention operations. The equivariant graph operation realizes the rotational equivariance and translational variability of the 3D graph coordinates, enabling the model to learn consistent feature representations in different molecular orientations. The global attention mechanism captures the global information of the molecule to obtain global features.

The content of the feature update in the equivariant graph operation is as follows:

$${H}^{(l)}={{MLP}}^{\left(l\right)}\left({H}^{\left(l-1\right)},{{||}{x}_{i}^{(l-1)}-{x}_{j}^{(l-1)}{||}}^{2}\right)$$

Among them, *H*^(*l*)^ is the node feature of the *l*-th layer, and ${x}_{i}^{(l-1)}$ is the node coordinate of node ${x}_{i}$ at the *l-*1-th layer.

The node update formula is as follows:

$${x}_{i}\leftarrow {x}_{i}+\Delta {x}_{i}$$

$$\Delta {x}_{i}={\sum}_{{j\epsilon} {{{\rm{N}}}}({{{\rm{i}}}})}{w}_{ij}({x}_{j}-{x}_{i})$$

Among them, ${x}_{i}$ is the node coordinate, $\Delta {x}_{i}$ is updated through the weighted sum of the relative positions between atoms, and ${w}_{ij}$ is the learnable weight based on the node and edge features. Ν(i) is the set of neighbors of node *i*.

The update of the attention layer is as follows:

$${H}^{(l)},{H}_{{cls}}={S{elf}A{ttention}{Layer}}^{\left(l\right)}({H}^{\left(l-1\right)},{H}_{{cls}})$$

$${A}_{{ij}}={Softmax}\left(\frac{Q{K}^{T}}{\sqrt{{d}_{k}}}\right)V$$

$${H}^{\left(l\right)}=A{V}_{{H}^{({{{\rm{l}}}}-1)}}$$

Among them, ${H}_{{cls}}$ is the entire molecular representation of the atom (bond) graph. Here, *Q, K, and V* are the Query, Key, and Value matrices, respectively, which are calculated through linear transformation. *A*_ij_ represents the attention intensity of node i towards node j. The number of heads in the *SelfAttentionLayer* is 4.

The atom graph has the advantages of being intuitive, computationally efficient, and rich in atomic features, which can represent the spatial arrangement and connection modes of atoms in a molecule. The bond graph, through its high-order structure, focuses more on reaction mechanisms and framework structures. A hybrid model of the two graphs is used to achieve multi-scale feature extraction. The alternating execution of equivariant graph operations and global attention operations ensures that the model is equivariant to rotation and translation while capturing the dependencies among all nodes in the molecule. The CLS feature is used as the global representation of the entire molecule.

### Statistics and reproducibility

All data analyses were conducted using Python 3.9 and Torch 2.7.1 software. To ensure the reproducibility of our findings, we provide detailed descriptions of the data generation process, including the sample sizes used in the synthetic data experiments. For experiments involving real-world datasets, we report the sources of the data, the preprocessing procedures, and all relevant analysis details. When comparing the outlier detection performance of MoleculeFormer against other models, we ran each model 10 times under fixed outlier ratios and recorded the ROC-AUC scores from each run. The final performance comparisons are based on the average AUC scores across runs to ensure robustness and reproducibility.

### Reporting summary

Further information on research design is available in the Nature Portfolio Reporting Summary linked to this article.

## Supplementary information

Supplementary Information

Reporting Summary

## Data Availability

The MoleculeNet benchmark library was downloaded from https://moleculenet.org/datasets-1. The phenotypic screening datasets were downloaded from https://github.com/idruglab/ChemBC. The ADME pharmacokinetic datasets were downloaded from https://github.com/molecularinformatics/Computational-ADME.

## Code availability

The codes for MoleculeFormer are freely available on GitHub (https://github.com/qinmingyuan-hub/molecular_graph) and on Zenodo (10.5281/zenodo.17346065)^60^.

## References

[1] Muratov, E. N. et al. QSAR without borders. *Chem. Soc. Rev.* **49**, 3525–3564 (2020). 32356548 10.1039/d0cs00098a PMC8008490

[2] Toropov, A. A. & Toropova, A. P. QSPR/QSAR: state-of-art, weirdness, the future. *Molecules*10.3390/molecules25061292 (2020). 10.3390/molecules25061292 PMC7143984 32178379

[3] Eklund, M., Norinder, U., Boyer, S. & Carlsson, L. Choosing feature selection and learning algorithms in QSAR. *J. Chem. Inf. Model.* **54**, 837–843 (2014). 24460242 10.1021/ci400573c

[4] Cao, D. S., Xiao, N., Xu, Q. S. & Chen, A. F. Rcpi: R/Bioconductor package to generate various descriptors of proteins, compounds and their interactions. *Bioinformatics* **31**, 279–281 (2015). 25246429 10.1093/bioinformatics/btu624

[5] Moriwaki, H., Tian, Y. S., Kawashita, N. & Takagi, T. Mordred: a molecular descriptor calculator. *J. Cheminform.* **10**, 4 (2018). 29411163 10.1186/s13321-018-0258-y PMC5801138

[6] Yap, C. W. PaDEL-descriptor: an open source software to calculate molecular descriptors and fingerprints. *J. Comput Chem.* **32**, 1466–1474 (2011). 21425294 10.1002/jcc.21707

[7] Duvenaud, D. K. et al. Convolutional networks on graphs for learning molecular fingerprints. *Adv. Neural Inf. Process. Syst.* **2**, 2224–2232 (2015).

[8] Muegge, I. & Mukherjee, P. An overview of molecular fingerprint similarity search in virtual screening. *Expert Opin. Drug Discov.* **11**, 137–148 (2016). 26558489 10.1517/17460441.2016.1117070

[9] Duda, R. O. & Hart, P. E. *Pattern Classification and Scene Analysis*, Vol. 3 (Wiley, New York, 1973).

[10] Sun, H. A naive Bayes classifier for prediction of multidrug resistance reversal activity on the basis of atom typing. *J. Med. Chem.* **48**, 4031–4039 (2005). 15943476 10.1021/jm050180t

[11] Cortes, C. & Vapnik, V. Support-vector networks. *Mach. Learn.* **20**, 273–297 (1995).

[12] Zhao, C. et al. Application of support vector machine (SVM) for prediction toxic activity of different data sets. *Toxicology* **217**, 105–119 (2006). 16213080 10.1016/j.tox.2005.08.019

[13] Zhou, J. et al. Prediction of molecular diffusivity of organic molecules based on group contribution with tree optimization and SVM models. *J. Mol. Liq.* **353**, 118808 (2022).

[14] Breiman, L. Random forests. *Mach. Learn.* **45**, 5–32 (2001).

[15] Cano, G. et al. Automatic selection of molecular descriptors using random forest: Application to drug discovery. *Expert Syst. Appl.* **72**, 151–159 (2017).

[16] Kang, B., Seok, C. & Lee, J. Prediction of molecular electronic transitions using random forests. *J. Chem. Inf. Model.* **60**, 5984–5994 (2020). 33090804 10.1021/acs.jcim.0c00698

[17] Babajide Mustapha, I. & Saeed, F. Bioactive molecule prediction using extreme gradient boosting. *Molecules* **21**, 983 (2016). 27483216 10.3390/molecules21080983 PMC6273295

[18] Wu, J. et al. Prediction and screening model for products based on fusion regression and xgboost classification. *Comput. Intell. Neurosci.* **2022**, 4987639 (2022). 35958779 10.1155/2022/4987639 PMC9357736

[19] Zagidullin, B., Wang, Z., Guan, Y., Pitkänen, E. & Tang, J. Comparative analysis of molecular fingerprints in prediction of drug combination effects. *Brief. Bioinform.*10.1093/bib/bbab291 (2021). 10.1093/bib/bbab291 PMC8574997 34401895

[20] Coley, C. W., Barzilay, R., Green, W. H., Jaakkola, T. S. & Jensen, K. F. Convolutional embedding of attributed molecular graphs for physical property prediction. *J. Chem. Inf. Model.* **57**, 1757–1772 (2017). 28696688 10.1021/acs.jcim.6b00601

[21] Kipf, T. N. & Welling, M. Semi-supervised classification with graph convolutional networks. *arXiv preprint arXiv:1609.02907* (2016).

[22] Velickovic, P. et al. Graph attention networks. *arXiv preprint arXiv:1710.10903* (2016).

[23] Hussain, M. S., Zaki, M. J. & Subramanian, D. Triplet interaction improves graph transformers: accurate molecular graph learning with triplet graph transformers. *arXiv preprint arXiv:2402.04538* (2024).

[24] Heid, E. et al. Chemprop: a machine learning package for chemical property prediction. *J. Chem. Inf. Model.* **64**, 9–17 (2024). 38147829 10.1021/acs.jcim.3c01250 PMC10777403

[25] Zhu, W., Zhang, Y., Zhao, D., Xu, J. & Wang, L. HiGNN: a hierarchical informative graph neural network for molecular property prediction equipped with feature-wise attention. *J. Chem. Inf. Model.* **63**, 43–55 (2022). 36519623 10.1021/acs.jcim.2c01099

[26] Wu, Z. et al. Hyperbolic relational graph convolution networks plus: a simple but highly efficient QSAR-modeling method. *Brief. Bioinform.* **22**, bbab112 (2021). 33866354 10.1093/bib/bbab112

[27] Cai, H., Zhang, H., Zhao, D., Wu, J. & Wang, L. FP-GNN: a versatile deep learning architecture for enhanced molecular property prediction. *Brief. Bioinform.*10.1093/bib/bbac408 (2022). 10.1093/bib/bbac408 36124766

[28] Satorras, V. G., Hoogeboom, E. & Welling, M. E(n) Equivariant Graph Neural Network. *arXiv preprint arXiv:2102.09844* (2024).

[29] Bolton, E. E., Wang, Y., Thiessen, P. A. & Bryant, S. H. in *Annual reports in computational chemistry* Vol. 4 217–241 (Elsevier, 2008).

[30] Rogers, D. & Hahn, M. Extended-connectivity fingerprints. *J. Chem. Inf. Model.* **50**, 742–754 (2010). 20426451 10.1021/ci100050t

[31] Durant, J. L., Leland, B. A., Henry, D. R. & Nourse, J. G. Reoptimization of MDL keys for use in drug discovery. *J. Chem. Inf. Comput. Sci.* **42**, 1273–1280 (2002). 12444722 10.1021/ci010132r

[32] Stiefl, N., Watson, I. A., Baumann, K. & Zaliani, A. ErG: 2D pharmacophore descriptions for scaffold hopping. *J. Chem. Inf. Model.* **46**, 208–220 (2006). 16426057 10.1021/ci050457y

[33] Landrum, G. *Fingerprints in the RDKit* (UGM, 2012).

[34] Gedeck, P., Rohde, B. & Bartels, C. QSAR− how good is it in practice? Comparison of descriptor sets on an unbiased cross section of corporate data sets. *J. Chem. Inf. Model.* **46**, 1924–1936 (2006). 16995723 10.1021/ci050413p

[35] Hall, L. H. & Kier, L. B. Electrotopological state indices for atom types: a novel combination of electronic, topological, and valence state information. *J. Chem. Inf. Comput. Sci.* **35**, 1039–1045 (1995).

[36] Ghose, A. K. & Crippen, G. M. Atomic physicochemical parameters for three-dimensional structure-directed quantitative structure-activity relationships I. Partition coefficients as a measure of hydrophobicity. *J. Comput. Chem.* **7**, 565–577 (1986). 10.1021/ci00053a005 3558506

[37] Wu, Z. et al. MoleculeNet: a benchmark for molecular machine learning. *Chem. Sci.* **9**, 513–530 (2018). 29629118 10.1039/c7sc02664a PMC5868307

[38] He, S. et al. Machine learning enables accurate and rapid prediction of active molecules against breast cancer cells. *Front. Pharmacol.* **12**, 796534 (2021). 34975493 10.3389/fphar.2021.796534 PMC8719637

[39] Fang, C. et al. Prospective validation of machine learning algorithms for absorption, distribution, metabolism, and excretion prediction: An industrial perspective. *J. Chem. Inf. Model.* **63**, 3263–3274 (2023). 37216672 10.1021/acs.jcim.3c00160

[40] Subramanian, G., Ramsundar, B., Pande, V. & Denny, R. A. Computational modeling of β-secretase 1 (BACE-1) inhibitors using ligand based approaches. *J. Chem. Inf. Model.* **56**, 1936–1949 (2016). 27689393 10.1021/acs.jcim.6b00290

[41] *AIDS Antiviral Screen Data*, http://wiki.nci.nih.gov/display/NCIDTPdata/AIDS+Antiviral+Screen+Data, accessed 2017-09-27.

[42] Martins, I. F., Teixeira, A. L., Pinheiro, L. & Falcao, A. O. A Bayesian approach to in silico blood-brain barrier penetration modeling. *J. Chem. Inf. Model.* **52**, 1686–1697 (2012). 22612593 10.1021/ci300124c

[43] *Tox21 Challenge*, http://tripod.nih.gov/tox21/challenge/, accessed 2017-09-27.

[44] Gayvert, K. M., Madhukar, N. S. & Elemento, O. A data-driven approach to predicting successes and failures of clinical trials. *Cell Chem. Biol.* **23**, 1294–1301 (2016). 27642066 10.1016/j.chembiol.2016.07.023 PMC5074862

[45] Kuhn, M., Letunic, I., Jensen, L. J. & Bork, P. The SIDER database of drugs and side effects. *Nucleic Acids Res.* **44**, D1075–D1079 (2016). 26481350 10.1093/nar/gkv1075 PMC4702794

[46] Rohrer, S. G. & Baumann, K. Maximum unbiased validation (MUV) data sets for virtual screening based on PubChem bioactivity data. *J. Chem. Inf. Model.* **49**, 169–184 (2009). 19434821 10.1021/ci8002649

[47] Mobley, D. L. & Guthrie, J. P. FreeSolv: a database of experimental and calculated hydration free energies, with input files. *J. Comput. Aided Mol. Des.* **28**, 711–720 (2014). 24928188 10.1007/s10822-014-9747-x PMC4113415

[48] Delaney, J. S. ESOL: estimating aqueous solubility directly from molecular structure. *J. Chem. Inf. Comput. Sci.* **44**, 1000–1005 (2004). 15154768 10.1021/ci034243x

[49] Mendez, D. et al. ChEMBL: towards direct deposition of bioassay data. *Nucleic Acids Res.* **47**, D930–D940 (2019). 30398643 10.1093/nar/gky1075 PMC6323927

[50] Fields, F. R. et al. Novel antimicrobial peptide discovery using machine learning and biophysical selection of minimal bacteriocin domains. *Drug Dev. Res.* **81**, 43–51 (2020). 31483516 10.1002/ddr.21601 PMC9202646

[51] Ye, Q. et al. Identification of active molecules against Mycobacterium tuberculosis through machine learning. *Brief. Bioinform.* **22**, bbab068 (2021). 33822874 10.1093/bib/bbab068

[52] Xiong, Z. et al. Pushing the boundaries of molecular representation for drug discovery with the graph attention mechanism. *J. Med. Chem.* **63**, 8749–8760 (2019). 31408336 10.1021/acs.jmedchem.9b00959

[53] Yang, K. et al. Analyzing learned molecular representations for property prediction. *J. Chem. Inf. Model.* **59**, 3370–3388 (2019). 31361484 10.1021/acs.jcim.9b00237 PMC6727618

[54] Chen, T. et al. Xgboost: extreme gradient boosting. *R. package version 0. 4-2* **1**, 1–4 (2015).

[55] Mullowney, M. W. et al. Artificial intelligence for natural product drug discovery. *Nat. Rev. Drug Discov.* **22**, 895–916 (2023). 37697042 10.1038/s41573-023-00774-7 PMC13118512

[56] Xiong, B. et al. Strategies for structural modification of small molecules to improve blood–brain barrier penetration: a recent perspective. *J. Med. Chem.* **64**, 13152–13173 (2021). 34505508 10.1021/acs.jmedchem.1c00910

[57] Brand, S. et al. Discovery of a novel class of orally active trypanocidal N-myristoyltransferase inhibitors. *J. Med. Chem.* **55**, 140–152 (2012). 22148754 10.1021/jm201091t PMC3256935

[58] Fushimi, M. et al. Discovery of potent, selective, and brain-penetrant 1 H-pyrazol-5-yl-1 H-pyrrolo [2, 3-b] pyridines as anaplastic lymphoma kinase (ALK) inhibitors. *J. Med. Chem.* **62**, 4915–4935 (2019). 31009559 10.1021/acs.jmedchem.8b01630

[59] Gunaga, P. et al. Selective I Kur Inhibitors for the Potential Treatment of Atrial Fibrillation: Optimization of the Phenyl Quinazoline Series Leading to Clinical Candidate 5-[5-Phenyl-4-(pyridin-2-ylmethylamino) quinazolin-2-yl] pyridine-3-sulfonamide. *J. Medicinal Chem.* **60**, 3795–3803 (2017). 10.1021/acs.jmedchem.6b01889 28418664

[60] Mingyuan, Q. *moleculeformer*10.5281/zenodo.17346065 (2025).

