# Supplementary material for: MoleculeFormer is a GCN-transformer architecture for molecular property prediction
Source: Commun Biol. 2025 Nov 25;8:1668. doi: 10.1038/s42003-025-09064-x (PMC12647147; doi:10.1038/s42003-025-09064-x)
Supplement: Supplementary file 1 — Supplementary Information [file 42003_2025_9064_MOESM1_ESM.pdf]

**Supplementary Table 1 Introduction to the atom encoding with a length of 136**

| | Encoding Method | Length | Notes |
|---|---|---|---|
| Atomic_number | One-hot | 101 | [Undefined, 0, … , 99] |
| Degree | One-hot | 7 | [Undefined, 0, 1, 2, 3, 4, 5] |
| Formal_charge | One-hot | 6 | [Undefined, -1, -2, 1, 2, 0] |
| Charity_type | One-hot | 5 | [Undefined, 0, 1, 2, 3] |
| Hydrogen | One-hot | 6 | [Undefined, 0, 1, 2, 3, 4] |
| Hybridization | One-hot | 6 | [Undefined, SP, SP2, SP3, SP3D, SP3D2] |
| IsAromatic | Boolean | 1 | |
| Atom_mass | Floating-point | 1 | |
| Atom_position | Floating-point | 3 | [x, y, z] |

**Supplementary Table 2 Introduction to the bond encoding with a length of 39**

| | Encoding Method | Length | Notes |
|---|---|---|---|
| Bond_type_atomPairs | One-hot | 25 | [Undefined, 'C-C', 'C-O', 'C-S', 'C-N', 'C-H', …] |
| Bond_type_Order | One-hot | 5 | [Undefined, SINGLE, DOUBLE, TRIPLE, AROMATIC] |
| IsInRing | One-hot | 3 | [Undefined, True, False] |
| Bond_center_position | Floating-point | 3 | [x, y, z] |
| Bond_direction | Floating-point | 3 | [x, y, z] |

**Supplementary Table 3 Numerical data for the anti-noise comparison shown in Figure 4**

| | | | | | | | | | | |
|---|---|---|---|---|---|---|---|---|---|---|
| MoleculeFormer | | | | | | | | | | |
| 10% | 0.7793 | 0.8022 | 0.8002 | 0.7810 | 0.7147 | 0.7855 | 0.7681 | 0.8404 | 0.8000 | 0.7973 |
| 15% | 0.7900 | 0.8109 | 0.7736 | 0.7895 | 0.7567 | 0.7351 | 0.7690 | 0.7682 | 0.7712 | 0.7708 |
| 20% | 0.7481 | 0.7740 | 0.7832 | 0.7705 | 0.7669 | 0.8034 | 0.7861 | 0.7810 | 0.6938 | 0.7862 |
| XGBoost | | | | | | | | | | |
| 10% | 0.7972 | 0.7842 | 0.7796 | 0.7885 | 0.7731 | 0.7752 | 0.772 | 0.7565 | 0.7881 | 0.7942 |
| 15% | 0.7711 | 0.7749 | 0.7605 | 0.7603 | 0.7485 | 0.7441 | 0.7428 | 0.7489 | 0.7599 | 0.7602 |
| 20% | 0.7528 | 0.7775 | 0.7253 | 0.7575 | 0.7257 | 0.7166 | 0.7229 | 0.74 | 0.7402 | 0.7486 |
| Attentive FP | | | | | | | | | | |
| 10% | 0.7986 | 0.7454 | 0.7654 | 0.7140 | 0.7885 | 0.7718 | 0.7549 | 0.7146 | 0.7070 | 0.7955 |
| 15% | 0.7856 | 0.7355 | 0.7873 | 0.7052 | 0.7361 | 0.7289 | 0.7305 | 0.6849 | 0.7743 | 0.7450 |
| 20% | 0.7334 | 0.7279 | 0.7284 | 0.7205 | 0.7055 | 0.7816 | 0.7093 | 0.6858 | 0.6896 | 0.7568 |
| FP-GNN | | | | | | | | | | |
| 10% | 0.6524 | 0.6919 | 0.7073 | 0.6980 | 0.6501 | 0.7202 | 0.7026 | 0.689 | 0.7461 | 0.7134 |
| 15% | 0.7190 | 0.6887 | 0.6941 | 0.6487 | 0.6889 | 0.7230 | 0.6906 | 0.6985 | 0.6743 | 0.7177 |
| 20% | 0.7177 | 0.6920 | 0.6443 | 0.6567 | 0.6659 | 0.6953 | 0.6506 | 0.6754 | 0.7106 | 0.6738 |
